# Supplementary material for: A fast, scalable and versatile tool for analysis of single-cell omics data
Source: Nat Methods. 2024 Jan 8;21(2):217–27. doi: 10.1038/s41592-023-02139-9 (PMC10864184; doi:10.1038/s41592-023-02139-9)
Supplement: Supplementary file 1 — Supplementary Fig. 1 and Supplementary Methods. [file 41592_2023_2139_MOESM1_ESM.pdf]

nature methods

Article https://doi.org/10.1038/s41592-023-02139-9

A fast, scalable and versatile tool for analysis of single-cell omics data

In the format provided by the authors and unedited

Supplementary Figures

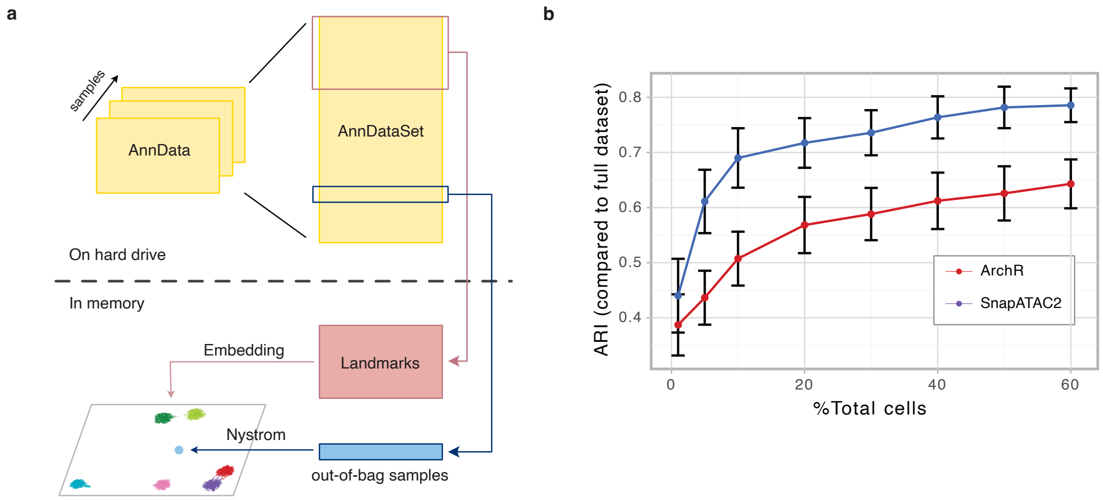

Supplementary Fig. 1 | SnapATAC2 overcomes memory constraints for analyzing atlas-scale datasets. **a**, Schematic representation of the out-of-core algorithm implemented in SnapATAC2 for processing datasets that surpass available memory capacity. **b**, Line plot comparing ARI scores when undergoing different levels of subsampling. ARI scores were calculated by comparing the embeddings generated from the complete dataset with those predicted using the subsampled dataset. Data are presented as mean values +/- SD across nine independent datasets. See Supplementary Table 3 for further details.

Supplementary Note 1: Supplementary Methods

SnapATAC2's data format. SnapATAC2's data format is based on the AnnData format[1], which is a versatile data structure and on-disk file format designed to facilitate the sharing of labeled data matrices. Despite its utility, the Python package "anndata"[1] has some shortcomings, particularly when operating in "backed mode". In this mode, the in-memory snapshot and on-disk data are not synchronized, causing unpredictable behavior. For example, only the "X" slot of the AnnData object is updatable, while other slots like "obs" remain unmodified on disk. Moreover, aside from the "X" component, all other elements are loaded into memory, which can be problematic for handling large datasets. To overcome these limitations, SnapATAC2 introduces its own out-of-core AnnData object with enhanced features. First, the AnnData object in SnapATAC2 is fully backed by its underlying HDF5 file, ensuring that any changes made in memory are immediately reflected on disk. Second, it employs lazy loading techniques to minimize memory consumption, allowing large files to be opened with almost no memory overhead. Additional features include an optional in-memory cache to speed up repeated access to data and an AnnDataSet object designed to lazily concatenate multiple AnnData objects, further enhancing its functionality and efficiency.

SnapATAC2's high performance preprocessing module. SnapATAC2's preprocessing module including BAM file processing, fragment file importing, QC metrics calculation, and cell by feature count matrix generation. All functions in this module are implemented using out-of-core algorithms, which enables SnapATAC2 to handle large-scale single-cell datasets efficiently without being limited by memory constraints.

Converting BAM files to fragment files. SnapATAC2 is capable of processing unfiltered BAM files, converting them into files containing the genomic coordinates of the fragments. A fragment record consists of five specific fields: the reference genome chromosome of the fragment, the adjusted start position of the fragment on the chromosome, the adjusted end position of the fragment on the chromosome, the cell barcode associated with the fragment, and the total number of read pairs linked to the fragment. In order to convert BAM files into fragment files, the following steps are taken. First, reads that are unmapped, not primary alignment, improperly aligned, have mapping quality less than 30, fail platform/vendor quality checks, or are optical duplicates are removed. Next, using out-of-core algorithms, the remaining reads are sorted by cell barcodes, and duplicate reads for each unique cell barcode are eliminated. Finally, the filtered and deduplicated BAM records are converted into fragments. This process is implemented by the "snapatac2.pp.make_fragment_file" function within the SnapATAC2 package.

Compute QC metrics. SnapATAC2 offers a comprehensive set of QC metrics to assess data quality, which include the number of unique fragments per cell, the proportion of mitochondrial reads per cell, the proportion of duplicate reads per cell, fraction of reads in peaks (frip) and the TSS enrichment score per cell. To calculate the TSS enrichment score, we first retrieved the TSS positions from a gene annotation file. We then aggregated Tn5-corrected insertions within a +/- 2000 bp range relative to each unique TSS (strand-corrected) across the entire genome. Subsequently, this profile was normalized based on the mean accessibility within the +/- (1900 to 2000) bp range from the TSS and smoothed at 11 bp intervals. The maximum value of the smoothed profile was taken as the TSS enrichment. The SnapATAC2 package's "snapatac2.pp.import_data" function facilitates this process.

Compute cell by feature count matrix. SnapATAC2 provides various methods to calculate the cell-by-feature count matrix. The "snapatac2.pp.add_tile_matrix" function computes the cell-by-bin matrix by dividing the genome into a fixed number of bins. The "snapatac2.pp.make_peak_matrix" function calculates the cell-by-peak matrix based on the peak file, while the "snapatac2.pp.make_gene_matrix" function computes the cell-by-gene matrix using the gene annotation file.

Doublet detection. We have adapted the Scrublet algorithm[2] to identify doublets in scATAC-seq data. To begin, we create simulated doublets by randomly pairing chromatin accessibility profiles of individual cells drawn from the existing scATAC-seq dataset. We then reduce the dimensionality of both original cells and simulated doublets using the spectral embedding algorithm in the SnapATAC2 package. Using these lower-dimensional embeddings, we train a k-NN classifier to distinguish between the simulated doublets and the authentic cells. This trained classifier produces a "doublet score" for each cell, quantifying the likelihood that it is a doublet. Notably, these doublet scores often display a bimodal distribution, signifying the existence of two distinct types of doublets: embedded and neotypic[2]. To translate these doublet scores into probabilities, we fit a Gaussian mixture model to their distribution. Each cell's doublet probability is then assigned based on its likelihood of falling into the mixture component with the higher mean, which corresponds to the doublet population. All these procedures are implemented in the "snapatac2.pp.scrublet" function available in the SnapATAC2 package.

Correction of Batch Effects. We modified a mutual nearest neighbor-based algorithm[3] to correct for batch effects in the data. To enhance computational efficiency, we first applied k-means clustering to each individual batch following dimensionality reduction. This yielded distinct clusters, for which we calculated centroids. We then identified pairs of mutual nearest centroids across batches, using them as anchor points to align cells from different batches and correct for batch effects. This methodology is adapted from previous work[3]. The procedure can be iteratively repeated to further refine the batch-effect correction, if necessary. The above steps are implemented in the "snapatac2.pp.mnc_correct" function from the SnapATAC2 package. We additionally provide "snapatac2.pp.harmony" and "snapatac2.pp.scanorama_integrate" functions to perform batch-effect correction using the Harmony[4] and Scanorama[5] algorithms, respectively.

Clustering. SnapATAC2 provides a range of scalable clustering algorithms such as k-means, DBSCAN, and Leiden clustering. Our benchmarks indicate that among these, Leiden clustering offers the highest accuracy for scATAC-seq data. To enhance the scalability of Leiden clustering, we employed approximate nearest neighbor search techniques to construct a k-NN graph based on the cells' low-dimensional embeddings. These procedures are incorporated into the "snapatac2.pp.knn" and "snapatac2.tl.leiden" functions within the SnapATAC2 software package.

Peak calling. In SnapATAC2, we employed the MACS2 "callpeak" command to identify peaks for each cell cluster, utilizing parameters such as "–keep-dup all –call-summits –nomodel –shift -10 –extsize 200". To generate a unified peak set, we aggregated peaks from all clusters and expanded the peak summits by 200 base pairs in both directions. To resolve overlapping peaks, we implemented an iterative removal strategy. Initially, the peak with the lowest p-value was retained, and any peaks directly overlapping with it were eliminated. This procedure was successively applied to the next most significant peak, and so forth, until all peaks were either preserved or removed due to overlap with a more significant peak. These procedures are incorporated into the "snapatac2.tl.call_peaks" function within the SnapATAC2 software package.

Differential accessibility analysis. To perform differential peak analysis between two sets of cells, we first calculated the fold change in chromatin accessibility for each peak. Peaks with fold changes below a user-defined threshold were subsequently filtered out. We then established two logistic regression models for each peak: a full model and a reduced model. The full model is described by $\log \frac{P_{\text{full}}}{1-P_{\text{full}}} = \beta_0 + \beta_1 r + \beta_2 c$, and the reduced model by $\log \frac{P_{\text{reduced}}}{1-P_{\text{reduced}}} = \beta_0 + \beta_1 r$, where P_{reduced} and P_{full} denote the probabilities of observing the peak under the full and reduced models, respectively. r represents the logarithm of the number of fragments, and c is a categorical variable indicating the cell set to which a given cell belongs. To assess the goodness-of-fit for these models, we utilized a likelihood ratio test to determine if the full model provided a significantly better fit than the reduced model and calculated the corresponding p-values. We further adjusted these p-values using the Benjamini-Hochberg method to control for multiple hypothesis testing. The model can be extended to include additional covariates, such as cell cycle phase, cell type, and batch. These steps are encapsulated in the "snapatac2.tl.diff_test" function within the SnapATAC2 software package.

Transcription factor motif enrichment analysis. To pinpoint transcription factors enriched in a specific set of peaks, we initially extract the genomic sequences corresponding to these peaks from the reference genome. Next, we search these sequences for transcription factor motifs by leveraging curated databases like CIS-BP[6]. We then calculate an enrichment score for each motif in the set of peaks as compared to a background dataset. This entire workflow is streamlined by the "snapatac2.tl.motif_enrichment" function available in the SnapATAC2 software package.

Regulatory network analysis. SnapATAC2 offers a suite of functions designed to construct regulatory networks from either scATAC-seq data or paired ATAC and gene expression profiles in single-cell multiome data. Initially, the "snapatac2.tl.init_network_from_annotation" function initializes a CRE-gene network by linking peaks to their closest genes based on a user-specified distance, using both a gene annotation file and a peak file. Following that, two functions, "snapatac2.tl.add_cor_scores" and "snapatac2.tl.add_regr_scores" are employed to assign regulatory scores to each edge in the network. The former calculates the correlation between a peak's accessibility and the expression of its corresponding gene across cells. The latter fits a regression model to this relationship, offering model choices that include elastic net and gradient boosting trees. Subsequently, the function "snapatac2.tl.add_tf_binding" queries transcription factor motif databases to identify transcription factors that bind to each peak, adding this information to the network. Finally, the "snapatac2.tl.link_tf_to_gene" function associates these transcription factors with their target genes based on the established regulatory network, using regression models to assign regulatory scores to these edges.

Removing memory constraints for atlas-scale datasets. SnapATAC2's matrix-free spectral embedding algorithm features linear space and time complexity, positioning it among the top-performing solutions. With 120 gigabytes of memory, SnapATAC2 can effectively perform dimensionality reduction for millions of cells. However, memory may still be a limiting factor when processing atlas-scale datasets, especially when combining data from multiple consortium studies or analyzing those with extremely high sequencing depth. To address this limitation, we developed an algorithm that circumvents the memory bottleneck of a computer, enabling the processing of massive numbers of cells in a reasonable timeframe (Supplementary Fig. 1a). Our approach involved creating an on-disk data structure for efficient storage and access to portions of the count matrix without loading the entire matrix into memory. We then performed matrix-free spectral embedding on a subsampled count matrix to obtain a reference embedding. Using this reference embedding, we applied the Nyström algorithm[7] to calculate the embedding for all cells without needing to ever store the complete count matrix in memory. Additionally, the final process can be distributed across multiple machines for a high degree of parallelism, enabling the processing of billions of cells relatively quickly. This feature is essential for analyzing large amounts of single-cell data generated from consortium efforts such as the BRAIN Initiative Cell Atlas Network (BICAN), the Human Cell Atlas (HCA), and the Human BioMolecular Atlas Program (HuBMAP).

Subsampling and approximation inherently lead to a reduction in accuracy. To evaluate this effect, we compared the embeddings generated from a subsampled dataset to those produced by the complete dataset. We discovered that our out-of-core algorithm yielded embeddings that were largely consistent with those generated by the complete dataset at various subsampling rates (Supplementary Fig. 1b and Supplementary Table 3). For comparison, we conducted the same analysis using the subsampled LSI algorithm implemented in ArchR. Our algorithm demonstrated greater accuracy across different subsampling rates compared to subsampled LSI (Supplementary Fig. 1b). For instance, at a 10% sampling rate, the average ARI score across nine experiments for the embeddings generated by our algorithm was nearly 0.7, while the average ARI score for those generated by subsampled LSI was about 0.5 (Supplementary Fig. 1b). This finding suggests that SnapATAC2 is more robust to subsampling approaches necessary for analyzing large-scale datasets.

References

1. Virshup, I., Rybakov, S., Theis, F. J., Angerer, P. & Wolf, F. A. Anndata: Annotated data. (2021).
2. Wolock, S. L., Lopez, R. & Klein, A. M. Scrublet: Computational identification of cell doublets in single-cell transcriptomic data. *Cell Systems* **8**, 281–291.e9 (2019).
3. Haghverdi, L., Lun, A. T. L., Morgan, M. D. & Marioni, J. C. Batch effects in single-cell RNA-sequencing data are corrected by matching mutual nearest neighbors. *Nature Biotechnology* **36**, 421–427 (2018).
4. Korsunsky, I. *et al.* Fast, sensitive and accurate integration of single-cell data with harmony. *Nature Methods* **16**, 1289–1296 (2019).
5. Hie, B., Bryson, B. & Berger, B. Efficient integration of heterogeneous single-cell transcriptomes using scanorama. *Nature Biotechnology* **37**, 685–691 (2019).
6. Weirauch, M. T. *et al.* Determination and inference of eukaryotic transcription factor sequence specificity. *Cell* **158**, 1431–1443 (2014).
7. Li, M., Lian, X.-C., Kwok, J. T. & Lu, B.-L. Time and space efficient spectral clustering via column sampling. in *CVPR 2011* (IEEE, 2011).
