# Supplementary material for: A fast, scalable and versatile tool for analysis of single-cell omics data
Source: Nat Methods. 2024 Jan 8;21(2):217–27. doi: 10.1038/s41592-023-02139-9 (PMC10864184; doi:10.1038/s41592-023-02139-9)
Supplement: Supplementary file 2 — Reporting Summary [file 41592_2023_2139_MOESM2_ESM.pdf]

nature portfolio

Corresponding author(s): Bing Ren

Last updated by author(s): Oct 26, 2023

Reporting Summary

Nature Portfolio wishes to improve the reproducibility of the work that we publish. This form provides structure for consistency and transparency in reporting. For further information on Nature Portfolio policies, see our Editorial Policies and the Editorial Policy Checklist.

Statistics

For all statistical analyses, confirm that the following items are present in the figure legend, table legend, main text, or Methods section.

n/a	Confirmed	
☐	☒	The exact sample size (*n*) for each experimental group/condition, given as a discrete number and unit of measurement
☐	☒	A statement on whether measurements were taken from distinct samples or whether the same sample was measured repeatedly
☐	☒	The statistical test(s) used AND whether they are one- or two-sided<br>*Only common tests should be described solely by name; describe more complex techniques in the Methods section.*
☐	☒	A description of all covariates tested
☐	☒	A description of any assumptions or corrections, such as tests of normality and adjustment for multiple comparisons
☐	☒	A full description of the statistical parameters including central tendency (e.g. means) or other basic estimates (e.g. regression coefficient) AND variation (e.g. standard deviation) or associated estimates of uncertainty (e.g. confidence intervals)
☐	☒	For null hypothesis testing, the test statistic (e.g. *F*, *t*, *r*) with confidence intervals, effect sizes, degrees of freedom and *P* value noted<br>*Give P values as exact values whenever suitable.*
☒	☐	For Bayesian analysis, information on the choice of priors and Markov chain Monte Carlo settings
☒	☐	For hierarchical and complex designs, identification of the appropriate level for tests and full reporting of outcomes
☒	☐	Estimates of effect sizes (e.g. Cohen's *d*, Pearson's *r*), indicating how they were calculated

Our web collection on statistics for biologists contains articles on many of the points above.

Software and code

Policy information about availability of computer code

Data collection	DuoClustering2018 (version 1.20.0)
Data analysis	We have used the following software packages for data analysis: SnapATAC2 (version 2.3.1), ArchR (version 1.0.1), Signac (version 1.6), Episcanpy (version 0.4.0), SCALE (version 1.1.2), PeakVI (version 0.19.0), scBasset (github: c15bec3a73fa1e04822db723338d234ca9d384ce), pycisTopic (github: 242c2a47aad475250f8abb2469a0e36085d6e460), SnapATAC (version 1.0), Higashi (github: 392da1d9cd7208aef0e8f6f7b1192a5aa0265ed2), scHiCluster (version 1.3.2), SCANPY (version 1.9.5), scvi-tools (version 1.0.3), mofapy2 (version 0.7.0), MIRA (version 2.1.0), Cobolt (version 1.0.1), scikit-learn (version 1.3.0), scib-metrics (version 0.3.3), scIB (version 0.3.3)<br>Custom code used for benchmark study: https://github.com/kaizhang/single-cell-benchmark.

For manuscripts utilizing custom algorithms or software that are central to the research but not yet described in published literature, software must be made available to editors and reviewers. We strongly encourage code deposition in a community repository (e.g. GitHub). See the Nature Portfolio guidelines for submitting code & software for further information.

March 2021

Data

Policy information about availability of data

All manuscripts must include a data availability statement. This statement should provide the following information, where applicable:

- Accession codes, unique identifiers, or web links for publicly available datasets
- A description of any restrictions on data availability
- For clinical datasets or third party data, please ensure that the statement adheres to our policy

We processed various public scATAC-seq datasets for our benchmarking analysis, with the datasets listed in Table 1. These include: 10x Genomics scATAC-seq data for brain (5k cells) and peripheral blood mononuclear cells (PBMCs, 10k cells), datasets from Chen et al., Ma et al., and Yao et al., available at this FTP server: http://ftp.cbi.pku.edu.cn/pub/glue-download/; Buenrostro2018 and synthetic scATAC-seq datasets, accessible on GitHub: https://github.com/pinellolab/scATAC-benchmarking; Human PBMCs data from GSE194122 (Gene Expression Omnibus (GEO)); Human cerebral cortex data from GSE162170 (GEO); Data from both human and mouse primary motor cortex, available from GSE229169 (GEO). Additionally, we utilized scHi-C datasets downloaded from this Google Drive link: https://drive.google.com/drive/u/0/folders/1j7ffz96kv_Ft3hicu2DRBmfcje0RA1sc, and scRNA-seq datasets obtained through the “DuoClustering2018” R package, which can be found on Bioconductor. Our study also includes single-cell multiome data for human PBMCs, downloaded from 10x Genomics: https://www.10xgenomics.com/resources/datasets/pbmc-from-a-healthy-donor-granulocytes-removed-through-cell-sorting-10-k-1-standard-2-0-0, as well as Paired-Tag data in mouse frontal cortex from GSE224560 (GEO). All the processed datasets generated in our study are made available as Anndata objects and can be accessed here: https://osf.io/hfs2v/.

Human research participants

Policy information about studies involving human research participants and Sex and Gender in Research.

Reporting on sex and gender	Not applicable
Population characteristics	Not applicable
Recruitment	Not applicable
Ethics oversight	Not applicable

Note that full information on the approval of the study protocol must also be provided in the manuscript.

Field-specific reporting

Please select the one below that is the best fit for your research. If you are not sure, read the appropriate sections before making your selection.

☒ Life sciences ☐ Behavioural & social sciences ☐ Ecological, evolutionary & environmental sciences

For a reference copy of the document with all sections, see nature.com/documents/nr-reporting-summary-flat.pdf

Life sciences study design

All studies must disclose on these points even when the disclosure is negative.

Sample size	The benchmark data were assembled to encompass a variety of experiment protocols, tissues, species, and data types, resulting in 20 unique datasets. When evaluating individual computational methods, we did not gather data from multiple runs since these methods exhibit minimal or no variation. Exceptions were made for benchmarking subsampling methods, where we used 9 samples, and for run time benchmarking, where 3 samples were taken. These sample sizes were selected to stabilize the variance.
Data exclusions	Data exclusions were carried out for the GSE194122 and Zemke_human datasets. Because in these cases, the data was generated using multiple donors or protocols, leading to pronounced batch effects. To ensure that our benchmark was not skewed by these batch effects, we opted to use only a subset of cells from these two datasets, specifically those originating from a consistent donor or protocol.
Replication	When benchmarking individual computational methods, we did not report data from multiple runs since these methods exhibit minimal or no variation. Exceptions were made for benchmarking subsampling methods, where we used 9 replicates, and for run time benchmarking, where 3 measurements were taken. All conclusions reported in this study are reproducible across at least 3 independent runs with different random seeds.
Randomization	In this study, there was no allocation of participants/samples/organisms into experimental groups. Instead, all computational methods were benchmarked uniformly across all datasets. Consequently, randomization was not a relevant consideration for our experimental design.
Blinding	Due to the nature of our study where all computational methods were benchmarked uniformly across all datasets, blinding was not necessary or applicable to our experimental design.

Reporting for specific materials, systems and methods

We require information from authors about some types of materials, experimental systems and methods used in many studies. Here, indicate whether each material, system or method listed is relevant to your study. If you are not sure if a list item applies to your research, read the appropriate section before selecting a response.

Materials & experimental systems

n/a	Involved in the study
☒	☐ Antibodies
☒	☐ Eukaryotic cell lines
☒	☐ Palaeontology and archaeology
☒	☐ Animals and other organisms
☒	☐ Clinical data
☒	☐ Dual use research of concern

Methods

n/a	Involved in the study
☒	☐ ChIP-seq
☒	☐ Flow cytometry
☒	☐ MRI-based neuroimaging
